# HLA-G peptide preferences change in transformed cells: impact on the binding motif

**DOI:** 10.1007/s00251-018-1058-2

**Published:** 2018-03-30

**Authors:** Alexander A. Celik, Gwendolin S. Simper, Wiebke Hiemisch, Rainer Blasczyk, Christina Bade-Döding

**Affiliations:** Institute for Transfusion Medicine, Hannover Medical School, Medical Park, Feodor-Lynen-Str. 21, 30625 Hannover, Germany

**Keywords:** HLA-G, Hodgkin’s lymphoma, Peptide presentation, Tumor immune escape

## Abstract

HLA-G is known for its strictly restricted tissue distribution. HLA-G expression could be detected in immune privileged organs and many tumor entities such as leukemia, multiple myeloma, and non-Hodgkin and Hodgkin’s lymphoma. This functional variability from mediation of immune tolerance to facilitation of tumor immune evasion strategies might translate to a differential NK cell inhibition between immune-privileged organs and tumor cells. The biophysical invariability of the HLA-G heavy chain and its contrary diversity in immunity implicates a strong influence of the bound peptides on the pHLA-G structure. The aim was to determine if HLA-G displays a tissue-specific peptide repertoire. Therefore, using soluble sHLA-G technology, we analyzed the K562 and HDLM-2 peptide repertoires. Although both cell lines possess a comparable proteome and recruit HLA-G-restricted peptides through the same peptide-loading pathway, the peptide features appear to be cell specific. HDLM-2 derived HLA-G peptides are anchored by an Arg at p1 and K562-derived peptides are anchored by a Lys. At p2, no anchor motif could be determined while peptides were anchored at pΩ with a Leu and showed an auxiliary anchor motif Pro at p3. To appreciate if the peptide anchor alterations are due to a cell-specific differential peptidome, we performed analysis of peptide availability within the different cell types. Yet, the comparison of the cell-specific proteome and HLA-G-restricted ligandome clearly demonstrates a tissue-specific peptide selection by HLA-G molecules. This exclusive and unexpected observation suggests an exquisite immune function of HLA-G.

## Introduction

Human leukocyte antigen (HLA)-G, a non-classical HLA class Ib molecule, plays an important role in immune protection and is in contrast to HLA class Ia molecules exclusively expressed at immune privileged sites (Kovats et al. 1990). Unlike for classical HLA class Ia alleles, only 58 alleles of *HLA-G* are described (Robinson et al. 2015), which result in even less protein isoforms. Among these, the HLA-G*01:01 allele is the most prevalent in different European populations (Castelli et al. 2014; Matte et al. 2000). Additionally, various splice variants of *HLA-G* can be processed (Hviid et al. 1998; Ishitani and Geraghty 1992) resulting in four membrane-bound forms (HLA-G1–G4) and three soluble forms (HLA-G5–G7). HLA-G1 constitutes the full-length HLA-G molecule that closely resembles other HLA class I molecules; however, alternative splicing leads to the exclusion of one or more α-domain in the HLA-G2, G3, or G4 isoforms. In addition to these, soluble isoforms corresponding to the membranous counterparts (e.g., HLA-G5 constitutes the soluble form of HLA-G1) are achieved by the retention of a stop codon after exon 4 (Dong et al. 2003; Gonen-Gross et al. 2005) or by cleavage of membranous HLA-G1 from the cell surface (soluble HLA-G1). However, while it is possible to detect these mRNA variants, the protein expression and biological function of these truncated forms remain elusive, while the HLA-G1, soluble HLA-G1, and HLA-G5 isoforms appear to be the most expressed forms in healthy tissue (Dahl et al. 2014; Paul et al. 2000). In the placenta (Kovats et al. 1990), immune tolerance is conferred by the expression of HLA-G on extra-villous trophoblasts (Apps et al. 2009) and through secretion of soluble HLA-G isoforms (Fournel et al. 2000). HLA-G interacts with different subsets of immune effector cells (NK, T, B, macrophages), usually resulting in inhibition of these cells (Bainbridge et al. 2000; Li et al. 2009; Naji et al. 2014; Rouas-Freiss et al. 1997). Inhibition of NK cell mediated cytolysis is facilitated through KIR2DL4 activation (Rajagopalan and Long 1999). T cell activity is inhibited by interaction of HLA-G with ILT-2 and ILT-4 (Riteau et al. 1999) and additionally leads to unresponsive and suppressive CD4^+^ T cell phenotypes (LeMaoult et al. 2004). Purified soluble HLA-G was shown to lead to apoptosis in activated CD8^+^ T cells (Fournel et al. 2000) and suppression of proliferation in CD4^+^ T cells (Bainbridge et al. 2000). Soluble HLA-G also interferes with immune regulation by induction of CD4^+^CD25^high^FOXP3^+^ Tregs (Selmani et al. 2008) and Tr1 cells; induction by HLA-G^+^ IL-10 producing DCs was shown in vitro (Gregori et al. 2010). In addition, it was shown that interaction of ILT-4 can also occur with β_2_m free forms of HLA-G (Gonen-Gross et al. 2005).

Structurally, HLA-G appears similar to other HLA class I molecules. The α1 and α2 domain form the peptide-binding cleft that presents self-peptides of preferably 9 amino acid (AA) residues in length (Diehl et al. 1996; Lee et al. 1995) to the immune system. HLA-G-restricted peptides feature anchor motifs at peptide position p2 in the form of Isoleucine (I) and Leucine (L), as well as Leucine (L) at pΩ with a strong Proline (P) auxiliary anchor at p3. X-ray analyses of peptide/HLA-G/β_2_m complexes showed that peptide presentation is facilitated analogous to other HLA class I proteins, although the peptide is positioned deeper in the cleft similar to the peptide presentation on HLA-E (Clements et al. 2005; Clements et al. 2007). Structural insight into KIR2DL4 complexed to HLA-G illustrated that Methionine^76^ (M^76^) and Glutamine^79^ (Q^79^) located in the α1 domain are critical for KIR2DL4 recognition, although specific binding sites of the receptor remain unknown (Yan and Fan 2005) and any potential peptide interactions remain intangible. In contrast, similar to the interaction of other KIRs with MHC-I, interaction between HLA-G and the ILT-2 or ILT-4 receptor is thought to be facilitated mainly through the α3 domain.

Hodgkin’s lymphoma (HL) is a malignant lymphatic disorder characterized by an abnormal type of mature B cells called Hodgkin-Reed-Sternberg (HRS) cells (Swerdlow et al. 2017). Classic HL (cHL) is designated by small amounts of HRS cells that are surrounded by normal immune cells. Similar to other B cell lymphomas, HRS cells tend to loose many of the typical B cell lineage factors and the expression of the B cell receptor (Kanzler et al. 1996; Schwering et al. 2003). Concurrently, expression of immune checkpoint proteins such as PD-L1 (Green et al. 2010; Roemer et al. 2016) and CD80 (Kosmaczewska et al. 2002; Murray et al. 1995) helps to evade immune recognition. At the same time, downregulation of HLA class I expression is a common feature especially in EBV-negative HL (Diepstra et al. 2008), thus preventing the presentation of neo-antigens. Studies on HRS cell lines performed by Liu et al. (2017) showed that each cell line affected overall HLA expression by mutations in either genes encoding for β_2_m, HLA-A, or the class II major histocompatibility complex transactivator (CIITA). Notably, lack of HLA surface expression should induce an NK cell response of the HLA class I^−^ HRS cells; however, the reactive infiltrate surrounding the cancer cells does not show increased presence of NK cells and absence of NK cell-mediated cytotoxicity. Potent inhibitors of NK cell activation include HLA class Ib molecules HLA-E and HLA-G. Indeed, the expression of HLA-G was suggested to be a potential immune evasion mechanism in HL. Diepstra et al. (2008) found HLA-G expression in 54% of HRS cells of lymphoma patients, and notably, HLA-G expression was associated with a lack of HLA class Ia surface expression. Further studies by Caocci et al. (2016) demonstrated that 55% of HRS cells from lymph nodes of cHL patients were positive for HLA-G expression. Interestingly, the surrounding lymphocytes and histiocytes were HLA-G positive and correlation with disease progression suggests an association of these immunoreactive patterns of HRS cells and the tumor microenvironment on disease outcome. Aberrant HLA-G expression was also described in other hematological malignancies such as non-Hodgkin lymphoma (Sebti et al. 2007), chronic lymphatic leukemia (Sebti et al. 2007), and multiple myeloma (Leleu et al. 2005) as well as in solid tumors, such as breast cancer (Konig et al. 2016; Rebmann et al. 2003), and non-small cell lung cancer, particularly in advanced disease stages (Ben Amor et al. 2016; Yan et al. 2015).

For HLA class Ia proteins, the variability of the heavy chain dictates the pathway of peptide recruitment and restricts the amount of presentable peptides from the available peptidome. The HLA class Ia molecule and/or the bound peptide together dictate the fate of a presenting cell. The nature of HLA-bound peptides influences the overall stability of a peptide-HLA complex (pHLA). Due to increased stability, prolonged surface expression of pHLA complexes implements enhanced T cell immunogenicity (Trujillo et al. 2014; Yewdell and Bennink 1999). The presented peptide repertoire is dependent on the specific HLA allotype and the available proteome, e.g., in HLA-B27 allotypes; it was suggested that depending on the subtype, differential interaction with tapasin (TPN) or the transporter associated with antigen processing (TAP) influences the presented peptide (Lopez de Castro et al. 2004), and in the case of HLA-B*44:28, it was shown that position 156 influences HLA/TPN association (Badrinath et al. 2012). However, of the potential tens of thousands of different pHLA combinations available in the cell, the presentation of certain self-peptides is favored (Hunt et al. 1992) and of these, only few are immunogenic (Assarsson et al. 2007; Harndahl et al. 2012). Wang et al. (1997) further showed that allogeneic responses against low-abundance epitopes are not limited by lower affinity of T cells for non-self MHC molecules, yet, the exchange of a single AA can dramatically alter the affinity of specific T cell receptors (Uchtenhagen et al. 2013).

The described differential immune functions of HLA-G make a biological sense of the HLA-G invariability doubtful and a variability and involvement of bound peptides obvious. HLA-G is non-polymorphic, however, able to differentiate in certain protein products dependent on the tissue where it is expressed. In this study, we focused on HLA-G*01:01 as the most prevalent allele in different populations and compared the peptide features after expression in two different cell types. We utilized the erythroleukemic HLA neg. cell line K562, a standard cell line for HLA allele-specific peptide determination and the cell line HDLM-2 that exhibits an HRS phenotype and is frequently used as an HL model cell line (Berglund et al. 2003) for the comparison of HLA-G tissue-specific peptide selection. The tissue specificity of peptides and the involvement of peptide sequences to the overall available surface for immune receptors seem distinct and a comprehensive analysis of HLA-G-restricted peptides indispensable. A deep insight into the biochemistry of pHLA-G complexes will guide towards understanding immune escape mechanism in tumors.

## Material and methods

### Antibodies

Anti-β_2_m (polyclonal, #A0072, Dako), anti-CRT (polyclonal #ABR-01176, Dianova), anti-ERp57 (polyclonal #ADI-SPA-585, Enzo Life Sciences), anti-HLA-A/B/C (W6/32, AbD Serotec®), anti-HLA-A/B/C-PE (W6/32, eBioscience), anti-HLA-G (MEM-G/9, Thermo Fisher Scientific), anti-TAP1 (polyclonal #ADI-CSA-620, Enzo Life Sciences), anti-TPN (polyclonal #ADI-CSA-625 J, Enzo Life Sciences), anti-V5 (MCA1360, ABD Serotec), rabbit anti-mouse IgG-HRP (polyclonal, #P0161, Dako), goat anti-rabbit IgG-HRP (polyclonal, #P0448, Dako), and rat anti-mouse IgG-PE (RMG1-1, Biolegend).

### Maintenance of cell lines

The cell lines HDLM-2, K562, and T2 were maintained in RPMI1640 (Lonza) supplemented with 10% heat inactivated FCS (Lonza), 2 mM L-glutamine (c. c. pro), 100 U/ml penicillin, and 100 μg/ml streptomycin (c. c. pro). For transduction, lentiviral particles were produced in HEK293T cells that were cultured in DMEM (Lonza) supplemented with 10% heat inactivated FCS, 2 mM L-glutamine, 100 U/ml penicillin, 100 μg/ml streptomycin, and 1 mg/ml Geneticin® (Life Technologies). Cell lines were maintained at 37 °C and 5% CO_2_.

### Cloning of HLA-G variants and generation of HLA-G expressing cell lines

Constructs encoding for full-length HLA-G*01:01 (exon 1–6) were generated from JEG-3 cDNA and subcloned into the lentiviral vector pRRL.PPT.SFFV.mcs.pre as previously described (Bade-Doeding et al. 2011). Site-directed mutagenesis was used to generate soluble sHLA-G*01:01 (exon 1–4) vectors as described previously (Celik et al. 2016). The respective inserts were verified through sequencing. Using the methods established by Bade-Doeding et al. (2011), K562 and T2 cells were stably transduced with full-length HLA-G*01:01, K562 and HDLM-2 cells were stably transduced with sHLA-G*01:01. Expression and efficiency of recombinant protein expression were confirmed by FACS, ELISA, or Western blot.

### Large-scale production of soluble HLA-G molecules and mass spectrometric analysis

Using soluble HLA technology (Kunze-Schumacher et al. 2014), sHLA-G*01:01 molecules were produced in bioreactors (Integra Biosciences), supernatant was harvested weekly, pooled, and filtered using a 0.45 μm membrane (Merck Millipore). After purification at pH 8.0 using *NHS-activated HiTrap* columns (Life Technologies) coupled to the HLA-G-specific monoclonal antibody MEM-G/9 or HLA class I specific monoclonal antibody W6/32, sHLA-G molecules were eluted (100 mM glycine/HCl buffer pH 2.7) from the column and the functionality of trimeric complexes verified using an W6/32-based ELISA. For separation of peptides from the complexes, 3 mg of each purified sHLA-G sample was treated with trifluoric acid (J. T. Baker) at a final concentration of 0.1% and separated from heavy chain and β_2_m using a 10 kDa cutoff membrane (Merck Millipore). The peptide solution was vacuum concentrated and treated with NH4HCO3, DTT, iodoacetamide, and trichloroacetic acid. After purification with 0.1% TFA on Pierce™ C18 Tips (Thermo Fisher Scientific), sample was eluted with 60% acetonitrile/0.1% TFA, vacuum-dried and solubilized in 30 μl 2% acetonitrile/0.1% TFA. Utilizing LC/MS (Dionex UltiMate 3000 high-performance LC system coupled to LTQ Orbitrap Velos Hybrid FT Mass Spectrometer), peptides were analyzed.

### Enzyme-linked immunosorbent assay

Ninety-six-well plates (NUNC Maxisorp) were coated with 500 μl/well W6/32 antibody in PBS overnight at 4 °C. After washing the plate 2× with 250 μl PBS + 0.05% Tween/well, the plate was blocked for 1 h with 250 μl/well with blocking solution (PBS containing 2% BSA *w*/*v*). The plate was loaded with protein standard and sample diluted in blocking solution and incubated for 2 h at RT. After washing the plate 4× with 250 μl PBS/T, 100 μl/well anti-β_2_m antibody was added. After 1 h incubation at RT, the plate was again washed 4×. One hundred microliter per well anti-rabbit HRP-conjugated antibody was added and incubated for 1 h at RT. The plate was washed 6× with PBS/T, and 100 μl TMB One™ substrate (KEM-EN-TEC Diagnostics) was added. The reaction was stopped using 100 μl/well acidic stop solution (3 M sulfuric acid, 1 M HCl in ddH_2_O) and the plate analyzed using a Synergy 2 ELISA microplate reader.

### Western blotting

Proteins were boiled at 95 °C for 15 min in SDS sample buffer containing reducing agent (Invitrogen by Life Technologies™) and separated on a 4–12% Bis-Tris gel (Invitrogen by Life Technologies™). After transfer to a PVDF membrane (Invitrogen by Life Technologies™), the membrane was blocked in 3% milk powder (Roth) in PBS (*w*/*v*). Incubation of primary antibodies was performed overnight at 4 °C. The next day, the membrane was washed 3× with PBS/T, incubated with secondary HRP-conjugated antibody for 1 h at RT, and finally washed again 3× with PBS/T. Clarity™ ECL substrate (Biorad) was added and the chemiluminescent signal detected using a FluorChem® FC2 imaging system.

### Immuno-precipitation of components of the peptide loading complex (PLC)

Immunoprecipitation experiments were performed essentially as described by Badrinath et al. (2012). Briefly, 1 × 10^7^ cells were lysed for 1 h in 400 μl lysis buffer, TBS containing 5 mg/ml digitonin (Sigma Aldrich) and protease inhibitors (Roche Diagnostics), on ice. The lysate was centrifuged at 16000×*g* for 15 min at 4 °C. The supernatant was precleared for 1 h at 4 °C with protein A sepharose (GE Healthcare) and end-to-end mixing and centrifuged for 1 min at 4600×*g* and 4 °C; a lysis control was set aside. This was followed by immunoprecipitation of proteins for 1 h at 4 °C with protein A sepharose coupled to 3 μg anti-HLA-G antibody. Subsequently, the samples were centrifuged for 1 min at 4600×*g* and 4 °C, the beads were washed 2× with 10 mM TrisHCL pH 7.4 containing 1 mg/ml digitonin and 450 mM NaCl followed by one washing step with 10 mM TrisHCL pH 7.4 containing 450 mM NaCl and one with 10 mM TrisHCL pH 7.4. Precipitated protein was eluted by boiling the samples in SDS buffer and used for final analysis.

### Analysis of HLA-G expression on transduced cells

5 × 10^5^ cells were washed 2× with 2 ml FACS buffer (PBS containing 2% FCS and 2 mM EDTA) at 500×*g* for 5 min at 4 °C. Samples were then incubated with 100 μl Fc-block (10% AB-serum in PBS) for 20 min at 4 °C followed by incubation with an anti-HLA-G antibody (MEM-G/9) directly in the Fc-block for 20 min at 4 °C. After washing 2× with 2 ml FACS buffer, the cells were incubated with fluochrome-labeled secondary antibody for 20 min at 4 °C and washed 2× with 2 ml FACS buffer. Finally, the cell pellet was resuspended in 250 μl buffer and analyzed using a BD FACSCanto™ II.

## Results

### HLA-G*01:01 restricted peptide features

**Fig. 1:**
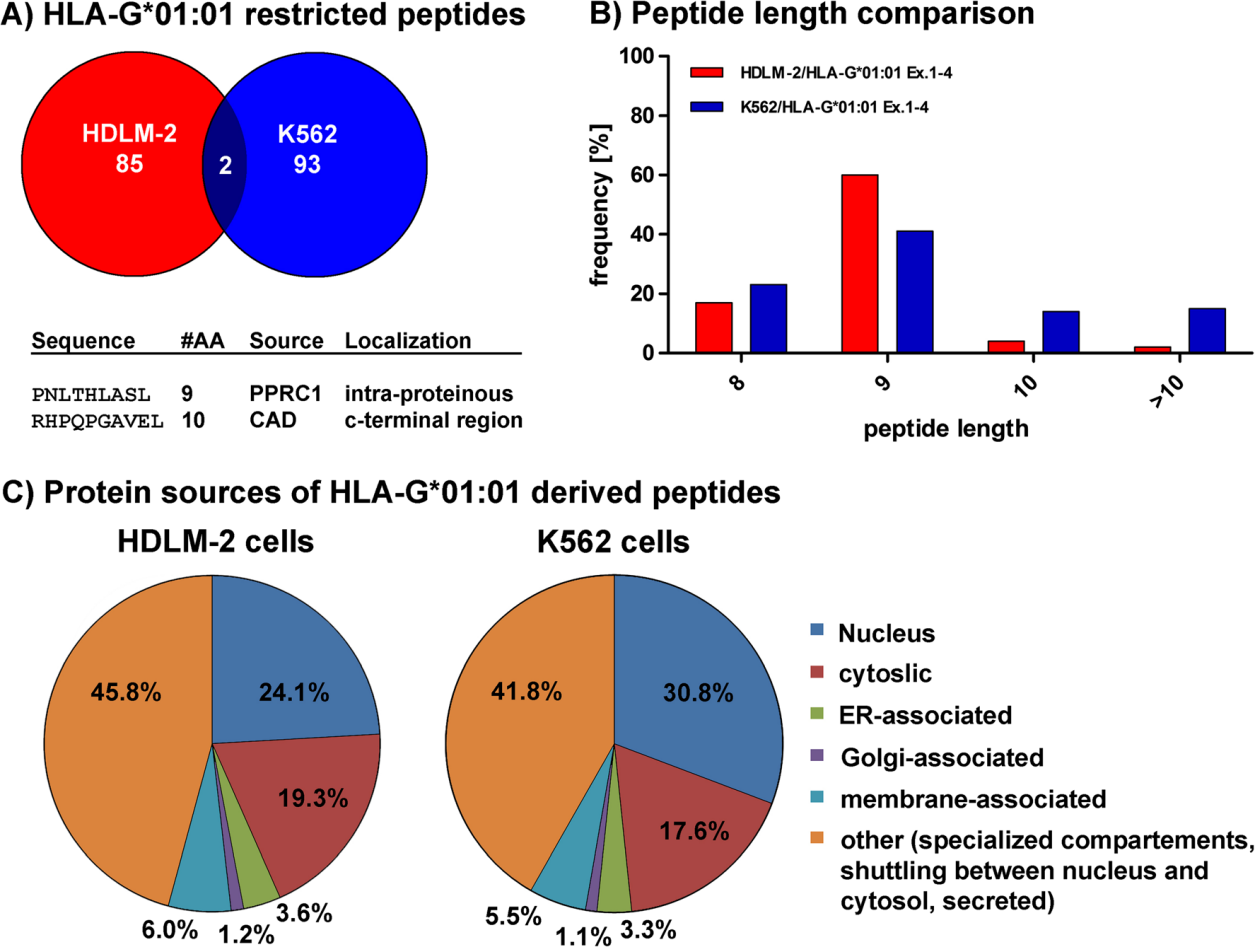
HLA-G*01:01-restricted peptide features. Peptides presented on HLA-G*01:01 were eluted and sequenced after expression in K562 or HDLM-2 cells. **a** Depicted is the total of individual, single peptide sequences from both cell lines. Between individual peptide sequences, overlap was observed in only two instances (PNLTHLASL, RHPQPGAVEL). **b** Depicted are the frequencies of the peptide length (number of AAs) that were observed. Most peptides showed canonical length of 9 AA, although longer peptides were also observed in fewer instances. Peptide frequencies are given on the y-axis; length of AAs is given on the x-axis. **c** Depiction of the localization of the protein source of the sequenced peptides. In both cell lines, peptides are recruited mostly from proteins shuttling between compartments, the nucleus and the cytosol

To determine the HLA-G*01:01 restricted peptide repertoire, HDLM-2 or K562 cells were stably transduced with sHLA-G*01:01 and, after cultivation in bioreactors, soluble HLA molecules were affinity purified. After isolation of HLA-G-restricted peptides, peptide sequences were analyzed using mass spectrometry. A total of 85 or 93 individual peptides were found in HDLM-2 or K562 cells (Fig. 1a), respectively. Only two shared peptides from K562 and HDLM-2 cells, respectively, could be detected, PNLTHLASL and RHPQPGAVEL (Fig. 1a). Peptides of 8 AA in length were observed in 17.0% (HDLM-2) or 23.2% (K562) of the cases; however, most of these peptides (Fig. 1b), 60.0% (HDLM-2) or 41.1% (K562), featured a length of 9 AA, while peptides of 10 AA in length could be virtually only detected in K652 cells (16.1%), constituting for only 2.4% in HDLM cells. Similarly, peptides longer than 10 AA were found in 17.1% (K562) or 1.3% (HDLM-2) of the cases. The protein source of most peptides from both cell lines derived from either nucleic proteins (24.1% HDLM-2, 30.8% K562), the cytosol (19.3% HDLM-2, 17.6% K562) or from proteins shuttling between the nucleus, and the cytosol or specialized compartments (45.8% HDLM-2, 41.8% K562, Fig. 1c). Compartment-specific peptides (ER, Golgi, membranes) were found in 6.0, 1.2, and 3.6% (HDLM-2) or 5.5, 1.1, and 3.3% (K562), respectively.

### The peptide anchor at p1 is tissue specific

**Fig. 2:**
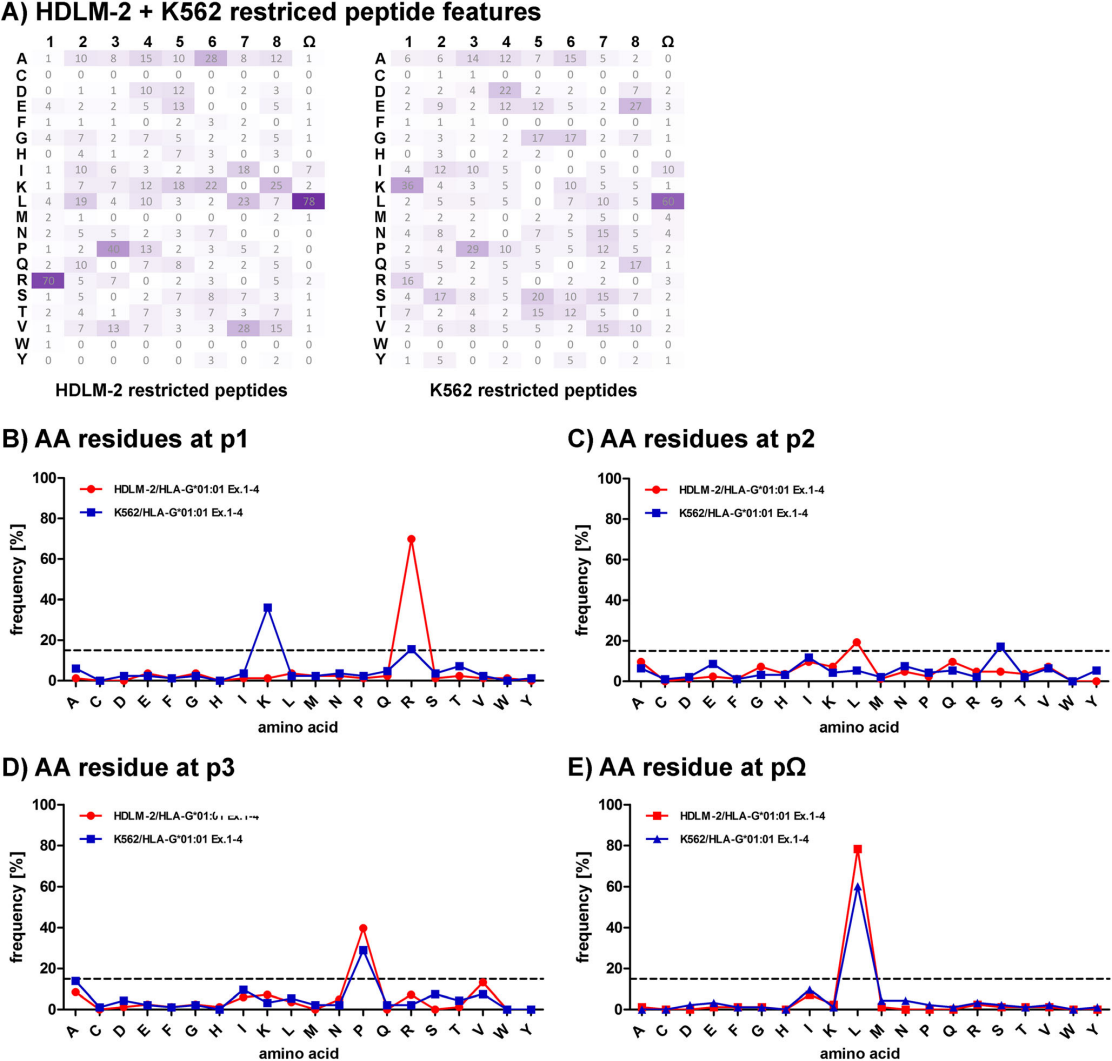
Difference in peptide features based on tissue derivation. **a** Frequencies of specific AAs at position 1 to 9 of identified 9-mers. AAs are given in one-letter code, higher frequency occurrences are depicted in more saturated color. **b**, **c**, **d**, **e** Depiction of the frequencies of AA residues at anchoring positions p1, p2, p3, and pΩ. AAs are given in one-letter code on the x-axis; frequencies are depicted on the y-axis. A 15% threshold is represented by a dashed line

Comparing the frequencies of individual AAs at every position in the sequences of identified 9-meric peptides reveals unexpected differences. An overview of AA frequencies at every peptide position (Fig. 2a) reveals a pattern that appears different at position p1 and p2, whereas at position p3 and pΩ, similarities are observed. All peptides are anchored at position p1, p3, and pΩ (Fig. 2b–e). A striking occurrence of Leucine (L) at pΩ could be observed with 78.3% for HDLM-2-derived peptides and 60.2% for K562-derived peptides. An anchor motif at p2 could not be defined (Fig. 2c). Noticeable peptides are anchored at p1 were furthermore a tissue-specific anchor motif could be observed; HDLM-2-derived peptides feature an Arginine (R) at p1 (69.9%) and K562-derived peptides a Lysine (K) (36.1%). A strong anchor motif at p3 Proline (P) for all peptides (39.8% HDLM-2, 29.0% K562) could be detected as previously specified (Diehl et al. 1996).

Due to the discrepancy in the HLA-G peptidome and peptide anchor motifs derived from the different cell lines, expression profiles of the respective proteins were analyzed using the mRNA expression data from the *protein atlas* (Thul et al. 2017). Certain HDLM-2-restricted proteins show even higher expression in K562 cells, yet peptides were exclusively identified to bind to HDLM-2-expressed sHLA-G molecules. For instance, the RIHDKAVAL peptide was identified solely from sHLA-G*01:01 molecules expressed in HDLM-2 cells; however, the source protein PDE4DIP shows mRNA expression levels that are elevated up to 16.7× in comparison to K562 cells. Further examples are given in Table 1.

**Table 1:** HDLM-2-specific HLA-G-bound peptides from proteins with increased expression in K562 cells and K562-specific HLA-G-bound peptides from proteins with increased expression in HDLM-2 cells

Sequence	#AA	Origin	Source	TPM↑ in K562
RGPPQRPKL	9	EIF4B	HDLM-2	2.1×
RIHDKAVAL	9	PDE4DIP	HDLM-2	16.7×
RAIQKKIDL	9	ERGIC3	HDLM-2	2.9×
RLKKSADTL	9	LDHA	HDLM-2	1.4×
Sequence	#AA	Origin	Source	TPM↑ in HDLM-2
KSPPPMNL	8	MEF2C	K562	2.7×
KYIHSANVL	9	MAPK3	K562	4.6×
KIIDSGPQL	9	FAM91A1	K562	3.5×
TAVISIGNQL	10	SYNE2	K562	24.0×

mRNA expression data from the *human protein atlas* (https://www.proteinatlas.org) (Thul et al. 2017). *TPM* transcripts per million, ↑ increase

### TAP independent peptide loading of HLA-G*01:01

**Fig. 3:**
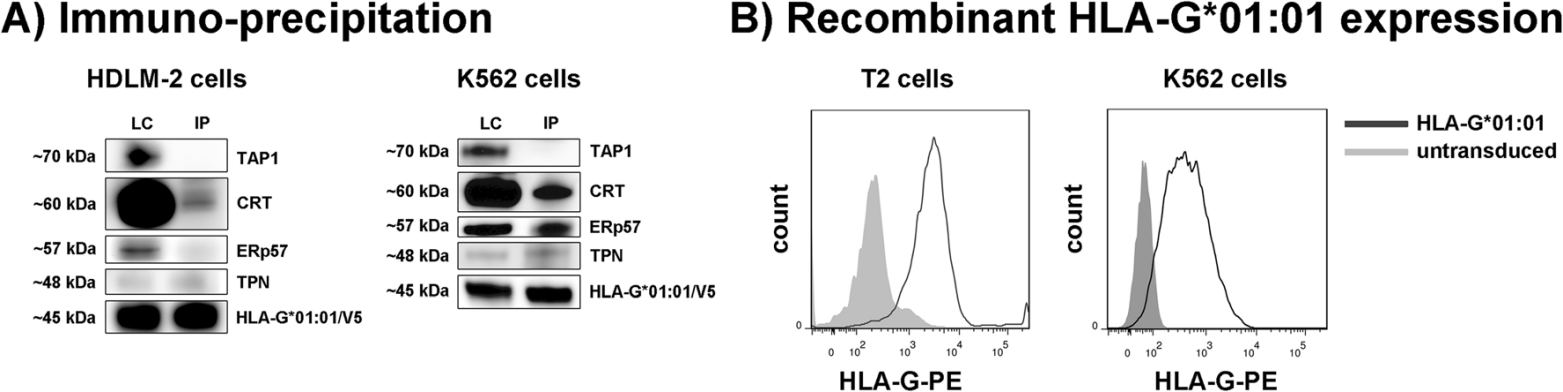
Expression of recombinant HLA-G molecules in HDLM-2 and K562 cells. **a** Immuno-precipitation of sHLA-G*01:01 was performed using an anti-HLA-G antibody (MEM-G/9) in HDLM-2 as well as K562 cells and Western blot against proteins of the PLC was performed. Notably, no association with TAP was observed. *LC* lysate control, *IP* immune-precipitation. **b** Using an anti-HLA-G-specific antibody (MEM-G/9), recombinant expression of HLA-G*01:01 on the surface of TAP-negative T2 could be detected

In both cell lines, virtually the same proteomic content is available; however, a diverging peptide anchor was observed for the same HLA-G*01:01 allele. This lead us to the speculation that maybe there are differences in the peptide selection and loading pathway. Therefore, the association of HLA-G with major proteins of the PLC was analyzed by immune-precipitation experiments using an HLA-G-specific antibody. Immuno-precipitation of sHLA-G*01:01 in K562 and HDLM-2 transfectants showed association with (CRT), ERp57, and TPN; however, no interaction with TAP could be detected (Fig. 3a). To assess whether HLA-G molecules are still present on the surface in the case of TAP deficiency, recombinant expression of HLA-G on the surface of TAP-deficient T2 cells was analyzed (Fig. 3b). The expression of HLA-G on the surface of these cells supported the observation that peptide loading of HLA-G occurs in the absence of TAP. The unconventional peptide loading pathway of HLA-G in both cell lines leads to the conclusion that the differential peptide repertoire might not be due to differential pathways.

## Discussion

HLA-G is a potent immune inhibitor and well known to induce tolerance during pregnancy (Hunt et al. 2006; Kovats et al. 1990). In contrast to HLA class Ia, expression of HLA-G is usually confined to the placenta and certain immune-privileged tissues; however, ectopic expression was found in many different tumor entities (Ben Amor et al. 2016; Leleu et al. 2005; Rebmann et al. 2003; Sebti et al. 2007) where it is thought to feature a tolerogenic effect on different subsets of immune cells including CD56^+^ NK cells, CD4^+^/CD8^+^ T cells, and B cells. Such non-reactive immune effectors are also present inside the reactive infiltrate surrounding HRS cells in cHL (Liu et al. 2014). HLA-G is discussed as an immune evasion mechanism in cHL (Caocci et al. 2016; Diepstra et al. 2008), since typically more than half of the HRS cells are positive for HLA-G expression, while HLA class Ia expression is downregulated at the same time appears feasible that HLA-G constitutes an immune evasion mechanism in cHL. The downregulation of HLA class Ia molecules is a meaningful immune escape strategy since the polymorphic nature of HLA class Ia molecules enables them to present a wide variety of peptides covering plenty of a pathogenic peptidedome (Wang et al. 2017). Additionally, through the limitation of HLA class Ib molecules to present a narrow variety of peptides effectively prevents the presentation of pathogenic ligands. In the case of HLA-E, for instance, presentation of peptides from HLA class I signal sequences (Braud et al. 1997) acts as a fall back mechanism to surveille HLA class I expression, yet, certain pathogens such as CMV use this system to further deceive immune effector cells from recognizing the lack of HLA class I expression during pathogenic episodes by providing suitable peptide analogues (Kraemer et al. 2014). However, we previously demonstrated the immunogenic capacity of CD56^+^ immune effector cells to distinguish between different peptide sequences and topologies when bound to HLA-E (Kraemer et al. 2015). Comparable peptide receptivity might be existing for HLA-G, since this HLA class Ib molecule possesses a marginal polymorphism but a wide range of immune features. Hence, the aim of the present study was to analyze differences in HLA-G peptide presentation based on the tissue where peptides are recruited from. We engineered sHLA-G molecules in two cell lines with differential proteomic background; both cell lines derived from leukemic cells and compared their peptide profiles. In general, the peptides presented by HLA class Ia molecules are restricted by the binding motif of the HLA subtype as well as the proteomic content available inside the cell and therefore in turn predetermine the available ligandome (de Verteuil et al. 2010; Fortier et al. 2008). Early peptide elution studies reported that HLA-G presents only a restricted set of peptides (Diehl et al. 1996); in term placenta, these are practically only derived from a cytokine receptor-like molecule (Ishitani et al. 2003). Apart from peptide invariance, HLA-G constitutes a classic peptide presenter presenting 9-mers as shown by pool sequencing studies of Diehl et al. (1996) and Lee et al. (1995). Most of the peptides we observed also displayed canonical length and were primarily derived from proteins available in the cytosol and the nucleus as well as proteins shuttling between these two compartments.

Considering the previously depicted conserved nature of HLA-G ligands, the variety of peptides and difference in the presented peptide repertoire from the two different sources, the erythroleukemic cell line K562 and the Hodgkin’s lymphoma cell line HDLM-2, was remarkable. The pΩ anchor Leu as well as Pro as an auxiliary anchor at p3 (Diehl et al. 1996) was described previously, although in both cases, p1 was divided between Arg and Lys (Lee et al. 1995) when expressed in LCL721.221 cells. We could identify both motifs; however, in the present study, the peptide anchor at p1 could be stringently attributed to the cell type the HLA-G molecules were expressed in. It is known for other class I alleles that peptide presentation is tissue specific (Fortier et al. 2008); however, anchoring positions are usually allele specific (Madden 1995; Yewdell and Bennink 1999). It is well known and for many HLA class Ia alleles established that allelic features dictate the peptide-binding motif. Therefore, a mismatch can be weighted by magnitude on the peptide motif. In the background of these HLA acknowledgements, it was unforeseeable that HLA-G features an alteration of the peptide motif depending on the tissue. The obvious question occurs whether the differential peptide motif arises by virtue of peptide availability in the different cell types. Hence, we analyzed the online available expression levels of protein sources from the *human protein atlas* (Thul et al. 2017) to define a tissue-specific ligandome. Most interestingly, we found that peptide selection and presentation by HLA-G*01:01 is not a matter of source availability, since K562 cells have the same or higher protein source availability for peptide presentation as HDLM-2 cells and vice versa. This strengthens the assumption that HLA-G selects and presents peptides of different anchor motifs tissue specifically. An explanation for the unexpected and unusual diverging peptide anchors for the same allele with virtually the same proteomic content might be the presence of different peptide selection and loading pathways. Therefore, we aimed to analyze the association of HLA-G*01:01 with major proteins from the PLC. Loading of optimized peptides is usually facilitated by the PLC. Here, TAP is important for peptide translocation and through association with TPN, TAP is allocated to the immediate vicinity of the PLC and thus the HLA molecule (Cresswell et al. 1999). This facilitates efficient loading of peptides that are 8–12 AA (Androlewicz and Cresswell 1996) in length and aides peptide optimization (Blum et al. 2013; Williams et al. 2002) by TPN. Nevertheless, it has been reported before that certain ER-derived antigens can be presented in the case of TAP impairment in mice and that these can present recognizable CTL epitopes (Durgeau et al. 2011). Additionally, it has been demonstrated that DCs are able to present antigens on MHC class Ia molecules TAP independently through an endolysosomal vesicular pathway, especially when stimulated by an TLR9 activator (Chen and Jondal 2009). In the present study, we could demonstrate that HLA-G*01:01 does not associate with TAP and additionally presents pHLA-G complexes on the surface of TAP-negative cells. A TAP-independent peptide loading might indicate a tissue-specific peptide selection of HLA-G, probably controlled through the presence of certain cytokines. Post-HLA class Ia downregulation and HLA class Ib upregulation, an increase in IL-10 production, could be traced in lung cancer (Urosevic et al. 2001). This is in line with findings that IL-10 expression is elevated in non-Hodgkin lymphoma (el-Far et al. 2004; Sebti et al. 2007) and findings that show that HLA-G expression is inducible by IL-10 (Moreau et al. 1999) in monocytes and human trophoblasts, highlighting the capabilities of prolonged HLA-G expression in immunosuppressive environments.

One of the limitations of our study is that it focused on the HLA-G*01:01 peptide presentation using transduced cell lines that does not take different HLA-G-specific regulatory factors into account. HLA-G transcription and also translation are known to be impacted by intron variations and variations in the UTR. Recently, Misra et al. (2014) observed a protective effect in end-stage renal disease (ESRD) and acute allograft rejection for HLA-G*01:01:01:01 and G*01:04:01 haplotypes whereas G*01:01:01:03, G*01:01:02, G*01:06, and G*01:05 N were risk associated. Additionally, higher levels of soluble HLA-G isoforms G5 and G6 were also present in ESRD cases suggesting that differences in the expression profile may modulate risk for ESRD and acute allograft rejection. Furthermore, findings by Agrawal et al. (2015) elucidated that certain HLA-G 5’URR SNPs increase the risk for idiopathic recurrent spontaneous abortion. Potentially, mutations that impact HLA-G transcript stability may also impact the generation of stable pHLA-G complexes. Additional studies, e.g., crystallographic analyses are further needed to aid understanding of the scope of such differential peptide presentations.

However, appreciating the results of peptide anchor alteration and tissue-specific peptide selection facilitates the understanding of the exquisite immune function of HLA-G and its amazing flexibility in the mediation of tolerance.
